# The Mere Exposure Effect in the Domain of Haptics

**DOI:** 10.1371/journal.pone.0031215

**Published:** 2012-02-08

**Authors:** Martina Jakesch, Claus-Christian Carbon

**Affiliations:** 1 Department of General Psychology and Methodology, University of Bamberg, Bamberg, Germany; 2 Faculty of Psychology, Department Basic Research, University of Vienna, Vienna, Austria; University of Regensburg, Germany

## Abstract

**Background:**

Zajonc showed that the attitude towards stimuli that one had been previously exposed to is more positive than towards novel stimuli. This *mere exposure effect* (MEE) has been tested extensively using various visual stimuli. Research on the MEE is sparse, however, for other sensory modalities.

**Methodology/Principal Findings:**

We used objects of two material categories (stone and wood) and two complexity levels (simple and complex) to test the influence of exposure frequency (F0 = novel stimuli, F2 = stimuli exposed twice, F10 = stimuli exposed ten times) under two sensory modalities (haptics only and haptics & vision). Effects of exposure frequency were found for high complex stimuli with significantly increasing liking from F0 to F2 and F10, but only for the stone category. Analysis of “Need for Touch” data showed the MEE in participants with high need for touch, which suggests different sensitivity or saturation levels of MEE.

**Conclusions/Significance:**

This different sensitivity or saturation levels might also reflect the effects of expertise on the haptic evaluation of objects. It seems that haptic and cross-modal MEEs are influenced by factors similar to those in the visual domain indicating a common cognitive basis.

## Introduction

Preferences play a major role in our lives: it starts before birth, when we are exposed to different kinds of odors that were shown to be decisive for later food preferences [1]. The discrimination between “good” and “bad” or “preferable” and “not preferable” is for sure not limited to odors and food preferences – people, potential mates, clothes, music, literature but also social situations or attitudes are examples for “categories” that can be preferable or not thus requiring decisions to be made.

Consequently, the preference formation in humans is of high interest in basic research as well as in applied research. The present study was conducted to investigate if haptic and cross-modal (haptic and vision) effects of mere exposure occur in the same manner as in the visual domain. This component of preference formation was in the focus of research relatively early, based on the mere familiarity of to be assessed categories. Hearing a well-known song from childhood days on the radio often leads to a warm and positive feeling. Already hundred years ago, Titchener [2] described the preference for familiar stimuli. The relationship between familiar stimuli and their higher preference ratings compared to novel ones was first investigated experimentally by Robert Zajonc [3]. By manipulating the exposure frequency (how many times a participant is exposed to specific stimuli), Zajonc [3] was able to show that preference ratings increased with higher exposure frequencies up to a point at which the ratings remained static or declined again. This is compatible with Fechner [4], who mentioned that in some cases (as compared to the “first impression” being the strongest impression) a repeated presentation of a stimulus is necessary to reach the full strength of impression. In a review, Bornstein [5] summed up twenty years of mere exposure (ME) research and reported essential factors and conditions under which ME effects occur weaker or stronger. Besides presentation variables (e.g., the number of exposures), measurement variables (e.g., delay between exposure and rating) and subject variables (e.g., personality and individual differences), two major stimulus variables were discussed: *stimulus type* and *stimulus complexity*. The effects of exposure seem to be closely related to *stimulus type*– the ME effect has been tested extensively using various visual stimuli (see [5]) with large effects sizes for polygons or meaningful words, but low effect sizes for drawings and paintings. Other studies used non-visual stimuli like music (e.g., [6], [7], [8], [9], [10], [11], [12]) or investigated olfactory or food preferences by using a ME paradigm [13], [14]. Subsuming, the most pronounced effects were found for stimuli that were novel or unfamiliar in the beginning – before the exposure starts. However, research on ME is sparse when it comes to tactile/haptic perception. To our knowledge, only one study published findings of visual and tactile effects of liking in a repeated exposure setting [15]. In the study, however, only two exposure frequencies were varied (novel versus once pre-viewed or pre-touched objects) so it cannot be qualified as a typical ME setting though the mean ceiling calculated in Bornstein's review used to be 20.95 exposures with a maximum ranging from 10 to 50 exposures [5]. Bornstein [5] also subsumed that in laboratory settings the highest affect or preference responses can be reached after only a small number of exposures. Thus, in the experiments reported in this paper, three exposure frequencies (F0 = novel stimuli, F2 = stimuli presented twice, F10 = stimuli presented ten times) are varied in a design similar to the one used in Zajonc, VanKrefeld, Tavris & Shaver's [16] experiments.

The second stimulus variable reported in Bornstein's [5] review, the *stimulus complexity*, seems to play a major modulating role in the process of exposure. In within-subjects designs, complex materials were rated as higher preferable than simple materials. Further, the “pleasantness” of simple stimuli mostly decreased with increasing familiarity, whereas high complex stimuli were rated as more pleasant at higher familiarity levels (e.g., [17]). As a consequence, in the present study we also varied stimulus complexity on two levels (simple vs. complex). Studies investigating visual, haptic and cross-modal (vision plus haptics) discrimination of shapes varying in scale and in their frequency of features, showed that the discrimination performance is dependent on the complexity level: visually, the stimulus set was discriminated successfully, when participants were asked to discriminate the same set haptically, losses of performance were reported for high levels of complexity [18]. Even though the discriminability is not a major variable in the present design, two separate pre-studies on complexity were performed to classify the complexity levels of our materials: one haptic pre-study, where our stimuli were rated according to their complexity under purely haptic conditions (results were used for the classification of complexity in Experiment 1) and a cross-modal pre-study, where participants rated the stimuli by using both percepts (vision plus haptics; results were used for the classification of complexity in Experiment 2).

The Mere Exposure (ME) effect was also often associated with implicit learning and memory due to the manipulation of familiarity. Based on this idea, several models tried to explain ME effects. An early approach was made by Berlyne [17], [19], [20] and Stang [21], [22], [23], [24], who proposed a two factor model of exposure effects: a combination of habituation and saturation effects expressed by an inverted u-shaped curve which represents increasing pleasure/preference until a peak is reached resulting in overexposure by further examination leading to a decline of pleasure/preference. Congruent with this view are the related findings for simple versus complex materials: for simple materials smaller numbers of exposures are necessary to reach the peak for overexposure or saturation as compared to high complex material. But the model has its limits: for instance the fact that unconscious learning effects cannot be explained. The reason for the ME effect to occur was discussed as an implicit learning process, for the effect behaves similar to implicit concept learning [25]. Further, ME effects do not seem to appear in childhood. Thus, Bornstein and D'Agostino [26] proposed a modified two factor model in which 1) the perceptual fluency increases through the repetitive exposure and 2) the feeling of familiarity based on a higher perceptual fluency of repetitive exposed stimuli leads to higher preference judgments. The construct of perceptual fluency is based on the assumption that familiar or previously experienced stimuli are easier “[…] to perceive, encode and process than are stimuli that have never seen before.” ([26], p. 105). For familiar objects more detailed perceptual representations are stored which enhances and speeds up the perceiving processes. Fang, Singh and Ahluwalia [27] compared the perceptual fluency theory with a further affect based fluency approach – the hedonic fluency model by Winkielman and Cacioppo [28] who argue that effects of processing fluency lead to positive affects that in turn influence the preference towards the familiar stimulus. Thus, instead of using cognitive input, the affect is used as information. Fang, Singh and Ahluwalia's [27] results suggest that for the evaluation either perceptual fluency or affective information is used when it is diagnostic. Oppenheimer [29] noted that fluency is a prominent cue that can be used in a variety of situations where judgments are needed. In this sense fluency does not only have a direct impact on the judgments but also an indirect one by changing the information that is stored as representation.

Ballesteros, Reales and Manga [30] gave evidence that mental representations of objects (familiar and unfamiliar/artificial) obtained by active touch are similar to those acquired by vision. A priming paradigm was used in the study to test fluency effects on encoding. Previously primed stimuli led to faster encoding effects also under haptic conditions. The authors were also able to reveal dissociation between implicit and explicit memory measures under haptic conditions. Their findings suggest that different object representations exist for both memory systems: an implicit memory test uncovered structural, shape-based representations, whereas an explicit memory test uncovered the recognition of low-level cutaneous information. These findings are comparable with proposed memory systems tested on the basis of visual and/or auditory input (e.g., [31]). The connection between mere exposure and learning and memory is a relevant one for the present study: it was shown that explicit *stimulus recognition* is not necessary to produce ME effects in the visual domain, moreover, in studies where stimuli were presented in a subliminal manner, the ME effect reached higher effect sizes than in the case of liminal/supraliminal stimulus presentation [5], [32]. One explanation for this phenomenon is related to Bornstein and D'Agostino's [26] model: if the reason for the “fluent” processing/encoding is obvious, a correction process starts and no or less misattribution towards a more positive attitude/higher preference might take place (e.g., [33]). Even though we are not able to present stimuli subliminally in the present study, there are indicators like the fluency effects in Ballesteros, Reales and Manga's [30] study or the findings of two exposures under haptic and cross-modal conditions in Suzuki and Gyoba [15] that ME effects could occur under haptic conditions in the same manner as under visual conditions, yet it was never tested in a classical ME setting.

The present study tested the exposure effect in a haptics only condition (only haptic input) plus in a cross-modal condition (haptic & visual input). Several studies showed that evaluative judgments are far more accurate when more sensory input is provided, for example: stimulus localization [34], slant estimation [35], and distance estimation [36] with better performance in cross-modal conditions compared to single modal conditions. In the present study, this modality based accuracy might be limiting the ME effect though richer representations might be formed enhancing the stimulus discriminability. Based on the fact that high *stimulus recognition* reduces the ME effects in the visual domain, we expect an attenuated effect in the cross-modal condition – hence the stimuli might be more recognizable in the cross-modal condition.

Haptic or touch-based preference evaluations are affected by interpersonal variables like, for instance, the participants' “Need for Touch” [37]. People considerably differ in their need for haptic or touch-based information in evaluative processes. Peck and Childers [37] differentiate between two factors of “Need for Touch” Scale (NFTS): the *autotelic* factor which represents hedonic-oriented responses and the *instrumental* factor which is goal-driven and intention based (e.g., intention to buy a specific product). Though we were interested in preferences – thus hedonic – oriented-judgments, the *autotelic* scale seemed to be more suitable to investigate possible influences through the participants' “Need for Touch” than the *instrumental* factor that predominately measures direct intentional consumer behavior. Hence, only the *autotelic* subscale was used in the current study.

### The present study

To sum up the aims of the present study: our aim was to measure the effects of different exposure frequencies (F0 = 0, F2 = 2, F10 = 10) on liking judgments of artificial objects under haptic and cross-modal conditions and to investigate factors modulating these effects (complexity, “Need for Touch”). The two experiments were designed specifically to investigate whether a) ME effects occur under a purely haptic condition (Experiment 1) in a similar manner as in studies with visual or auditory stimuli which might indicate that “haptic memory” is based on similar mechanisms like “visual/auditory” memory; b) if a cross-modal condition (Experiment 2) leads to attenuated effects based on a correction of judgments through a richer representation, c) if comparable modulating effects of complexity under the haptic condition can be found as reported for visual and/or auditory stimuli (Experiment 1 and Experiment 2) and lastly d) if the “Need for Touch” of the participants modulates the results – “experts” in touch could on the one hand show stronger ME effects based on their expertise in touching in general or might be less affected by the familiarity-based fluency.

### Materials and Methods Experiment 1 ME – “haptics only”

#### Participants

Sixty undergraduate students with a mean age of 21.8 years (35 female, 25 male) participated for course credit. All participants had normal or corrected-to-normal visual acuity and normal tactile sensitivity.

#### Materials

The Semmes–Weinstein Monofilaments and the Two Point “Disk–Criminator” tests were used to measure tactile sensitivity. Participants' visual acuity was measured with the Oculus © Low–Vision Test (viewing distance = 40 cm). This test includes seven short texts of different font sizes and a chart with *Snellen E* and *Landolt C optotypes*. Participants' “Need for Touch” was measured using the same named scale. The scale consists of six items, for example: “Touching products can be fun” ([37], p. 432).

#### Stimuli

**Figure 1:**
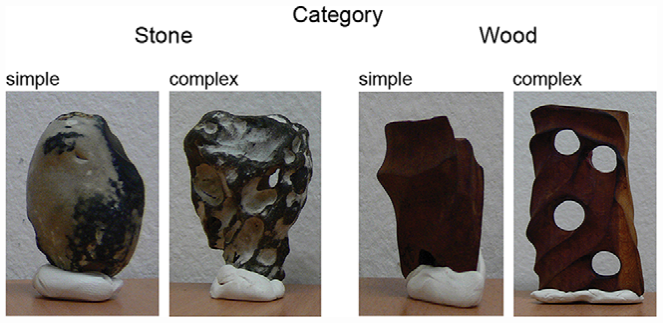
Examples of objects used in the Experiments. Note: The white base was added only for the photographic documentation.

Twenty-four artificial objects (12+12 pieces of wood+stone) were used in the present study. Artificial stimuli were chosen in order to hold the novelty level constant for all our participants so that these materials provide an optimal base line for our familiarity manipulation. The stimuli were weighted and their size was measured to control for possible effects of weight and size. The weight of the wooden stimuli ranged from 1 to 9 g, mean size (length×height×width) = 5.8 cm×1.5 cm×2.7 cm; the weights of the stone stimuli ranged from 7 to 27 g, mean size (length×height×width) = 3.7 cm×1.9 cm×2.7 cm. In the later analyses, the influence of size and weight parameters was controlled, statistically. Further, the complexity of each object was measured on a 7-point scale (1 = simple; 7 = complex) in two pre-studies either haptically or cross-modally (see Figure 1) to categorize the materials in two complexity classes (simple versus complex).

#### Procedure

**Figure 2:**
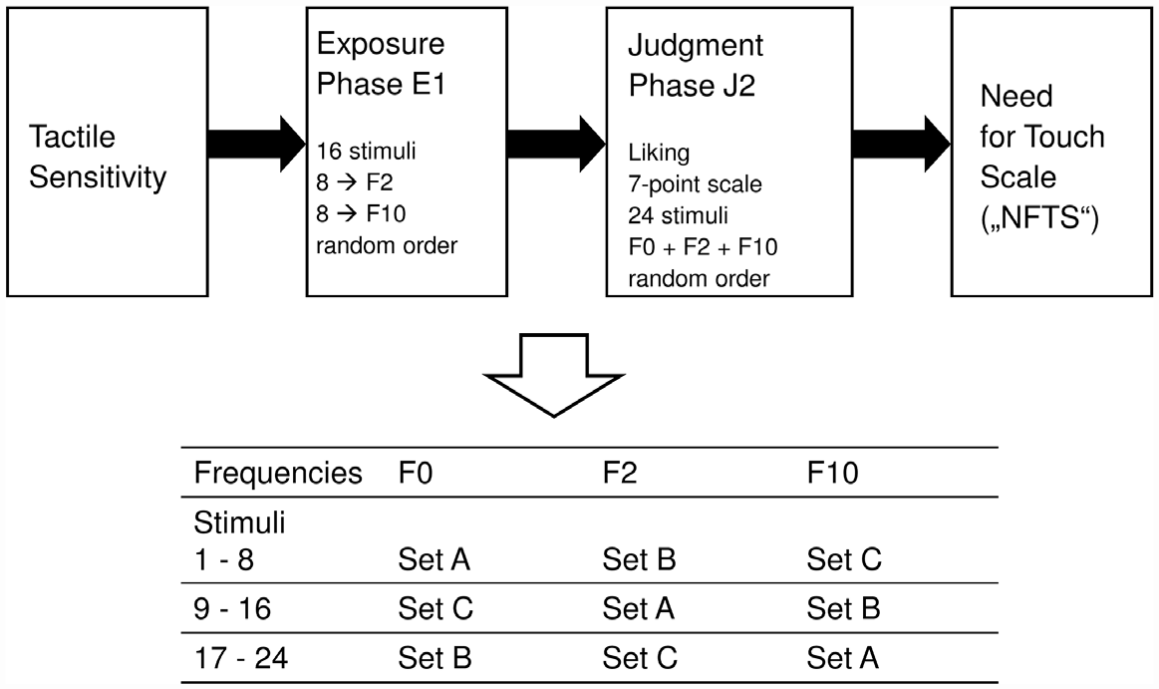
Schematic procedure of the whole experimental session plus balancing procedure for frequencies and stimuli. Participants were assigned randomly to one of the three sets.

Participants were tested individually. The design of the current study was adopted from Zajonc, VanKreveld, Tavris and Shaver [16]. Three sets (A, B, C) of stimuli (each consisting of eight stimuli, four made of wood and four made of stone) were created. The exposure frequency was varied on three levels pre evaluation: F0 = novel stimuli; F2 = stimuli presented twice and F10 = stimuli presented ten times. Sets and frequencies were counterbalanced between participants. In Figure 2, the balancing procedure (stimuli×exposure frequencies) is displayed in detail. Participants were randomly assigned to one of the sets. All tests (tactile sensitivity/visual acuity) were conducted in the beginning of each experimental session. Participants were then blindfolded (by using a mask) and were also told to avoid touching anything but the stimuli so that their ratings would not be influenced by other surfaces. In the “exposure phase” (Phase E1), two of the three sets of stimuli were presented twice (F2) and ten times (F10). During the subsequent “judgment phase” (Phase J2), all stimuli had to be rated on a 7-point scale (1 = “I do not like it at all”; 7 = “I like it very much”). In both phases, the stimuli were placed inside the hands of the participants in randomized order to be explored actively. After the experiment had ended participants were fully informed about the study and allowed to ask questions. Written consent was obtained from each participant prior to the experimental session. As all data were collected anonymously and no harming procedures were used, ethical approval was not sought for the execution of this study.

### Results Experiment 1

Two repeated measures ANOVAs were calculated with the within-subjects factor *exposure frequency:* 1) by stimuli to control for weight as well as for size and to add *complexity* (simple versus complex) and *stimulus category* (stone versus wood) as between factors and 2) by participants to investigate possible interpersonal differences. The *autotelic* “NFTS” score (median split for high versus low) was set as between factor.

#### Analysis by stimuli

**Figure 3:**
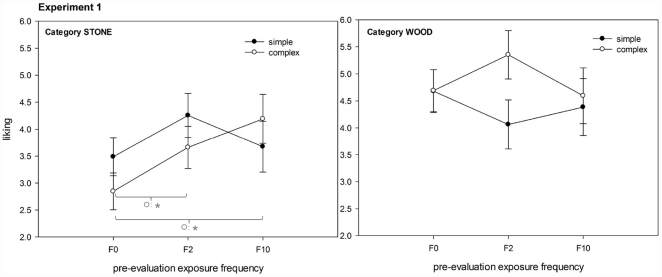
Analysis by stimuli: effects of pre-evaluation exposure frequency (F0, F2, F10) on liking split by category and complexity. Left graph: category *stone*; right graph: category *wood*.

Effects of *exposure frequency* were found *F*(2, 16) = 5.15, *p* = .02, *η*_p_^2^ = .39, as well as a three-way interaction between exposure frequency * stimulus category * complexity, *F*(2, 16) = 9.50, *p*< = .01, *η*_p_^2^ = .53. The effects of exposure frequency were specifically found for high complex stimuli with significantly increasing liking from F0 to F2 and F10, but only for the stone category (see Figure 3).

#### Analysis by participants

**Figure 4:**
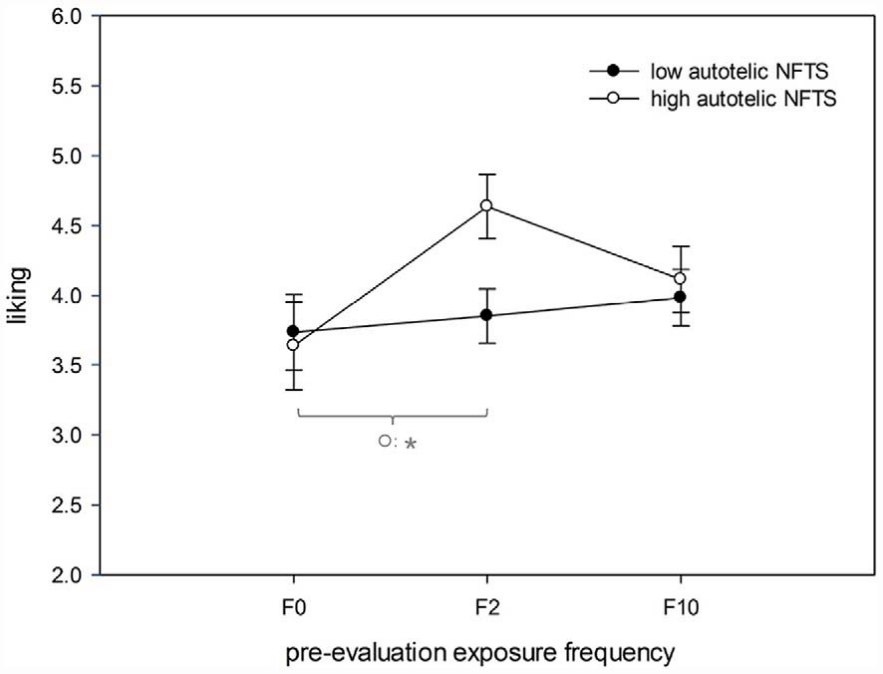
Analysis by participants: effects of pre-evaluation exposure frequency (F0, F2, F10) on liking split by autotelic Need for Touch Scores (NFTS).

Significant effects were found for exposure frequency, *F*(2, 23) = 4.62, *p* = .02, *η*_p_^2^ = .29, and the two-way interaction between exposure frequency * autotelic, *F*(2, 23) = 3.60, *p* = .03, *η*_p_^2^ = .24. These findings indicate that exposure effects are modulated by the participants “Need for Touch” though differences only occurred within the group of participants with a high “Need for Touch” (see Figure 4).

It seems that the ME effect is also stable and replicable in the haptic domain – with varying effects for the stimulus category. As the stimuli in the category stone are more similar within the category compared to the wooden stimuli, the lack of ME effects in the category wood might be due to a higher stimulus discriminability. If this is the case, the participants might have been aware of the objects' repetition and thus attributing the fluency effect to the exposure frequency in accordance with the correction processes discussed by Bornstein and D'Agostino [26] or Lee [33]. Through additional visual information, this effect should be more pronounced and probably also influencing the results in the category stone. Experiment 2 was planned to control for this effect.

### Materials and Methods Experiment 2 ME – “haptics plus vision”

In order to investigate cross modal ME effects, a second experiment was conducted using the same design and stimuli as in Experiment 1. Participants were able to see and touch the stimuli compared to Experiment 1 where participants could only inspect the material haptically without vision.

#### Participants

Thirty undergraduate students with a mean age of 24.7 years (25 female, 5 male) participated for course credit. All participants had normal-or-corrected to normal visual acuity and normal tactile sensitivity and were not tested in Experiment 1.

#### Materials

The same tests as in experiment 1 were used to measure tactile sensitivity (Semmes-Weinstein Monofilaments; Two Point “Disk–Criminator”) and visual acuity (Oculus © Low–Vision Test; viewing distance = 40 cm). Again, the “Need for Touch” scale was presented at the end of the experimental session [37].

#### Stimuli

The same twenty-four objects (12+12 pieces of wood and stone) were used as in Experiment 1. The complexity of each object was measured on a 7-point scale (1 = simple; 7 = complex) in two pre-studies either haptically or cross-modally.

#### Procedure

The procedure, including the “exposure phase” and the “judgment phase”, was identical to Experiment 1. Again, the participants' “Need for Touch” was measured in order to investigate interpersonal differences.

### Results Experiment 2

Similar to Experiment1, two repeated measures ANOVAs were calculated with the within-subjects factor *exposure frequency:* 1) by stimuli to control for weight and to add *complexity* (simple versus complex) and *stimulus category* (stone versus wood) as between factors and 2) by participants to investigate possible interpersonal differences. The “NFTS” Score = *autotelic* (high versus low) was set as between factor.

#### Analysis by stimuli

**Figure 5:**
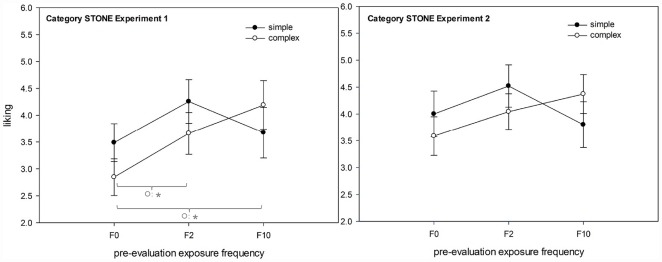
Comparison of analysis by stimuli: effects of pre-evaluation exposure frequency (F0, F2, F10) on liking in Experiment 1 (left) and in Experiment 2 (right) in the category stone.

No significant effect was found for the main factor *exposure frequency*, *F*(2, 16) = 1.20, *p* = .31, *n.s.* The three-way interaction between *exposure frequency* * *stimulus category* * *complexity* showed a trend towards significance, *F*(2, 16) = 3.16, *p* = .06, *η*_p_^2^ = .28, marginal trend, indicating a similar pattern as in Experiment 1. Figure 5 shows a comparison between Experiments 1 and 2.

#### Analysis by participants

**Figure 6:**
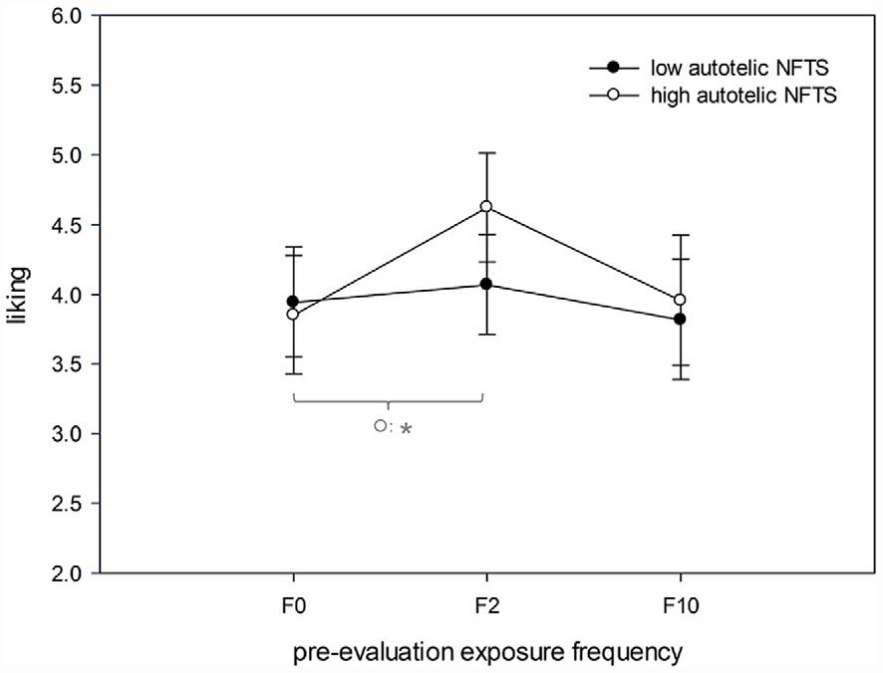
Analysis by participants: effects of pre-evaluation exposure frequency (F0, F2, F10) on liking split by the autotelic Need for Touch Scores (NFTS).

Across participants, a trend to significance was found for *exposure frequency*, *F*(2, 23) = 3.37, *p* = .05, *η*_p_^2^ = .23. The two-way interaction between *exposure frequency* * *autotelic* revealed no significant effect, *F*(2, 23) = 1.36, *p* = .26, *n.s.* (see Figure 6).

Similar to the “haptics only” condition in Experiment 1, effects of exposure frequency were found within the group of participants with a high “Need for Touch”. Further, the additional visual input appears to be overwriting the exposure effects even though the data pattern shows a similar direction compared to the haptics condition. The cross-modal input seems to result in a more detailed representation of the stimuli, which might evoke a correction process of familiarity-based fluency.

## Discussion

As the mere exposure (ME) paradigm is a valuable method to test familiarity-based preferences, the current study aimed to extend previous visual- and auditory-based results to the domain of haptic perception. In order to investigate haptic and cross-modal ME effects, two experiments were conducted. Experiment 1 tested whether ME effects occur under purely haptic investigation conditions in a similar manner as in studies, which used visual or auditory stimuli. Experiment 2 tested if a cross-modal condition leads to attenuated effects based on a correction of judgments through a richer representation.

The general aim was to compare modulating effects of complexity in the haptic condition with previous findings for visual and/or auditory stimuli. Specifically, if there are hints for a common cognitive basis – e.g. that “haptic memory” is based on similar mechanisms like “visual/auditory” memory – for ME effects in the visual and the haptic domain. Further, the influence of interpersonal differences (in the current study the “Need for Touch” of the participants) was tested.

The present study has revealed several results: first and most importantly, we were able to show the first time Mere Exposure effects based on an experimental variation of exposure frequency (F0, F2 and F10) in the domain of haptics. The classical increase of preference with higher exposure frequency was reported for high complex materials (category stone). The result is limited to the stimulus category stone as we were not able to show ME effects for the stimulus category wood. The fact that the exposure effects occurred in the category stone can only be explained by the stimulus discriminability. Zajonc et. al. [16] noted that the exposure effects are mostly true for stimuli which cannot be discriminated easily. The wooden stimuli in our set presumably varied stronger in their appearance than the stone stimuli. Therefore, the recognition for stimuli in the category wood might have been extensively higher which could have led to a correction process of the perceived fluency as described by Lee [33] or Bornstein and D'Agostino [26]. Instead of attributing the effects of fluency towards a more positive attitude for the repeated stimuli, the participants might have been aware of the repetition and thus corrected their judgments of preference.

Second, the attenuated effects for the cross-modal condition in Experiment 2 might be also due to that mechanism: through the additional sensory input, richer representations might have been formed and stored so that the stimuli in the category stone reached a higher level of discriminability/recognisability. An alternative explanation stems from Whittlesea and Price [38] who argued that not necessarily different memory resources are the reason for the dissociation between higher preferences for familiarized materials and stimulus recognition. If a stimulus recognition task was announced, in most cases no ME effects were found, whereas on the other hand if preference judgments were used as dependent variable, often only a chance level of stimulus recognition was measured. Some researchers argued that different memory systems are used: for stimulus recognition the explicit memory is used, whereas implicit memory processes take place when preferences are measured [26], [39]. Whittlesea and Price [38] claim that simply different strategies are used to gather relevant information about objects depending on the task. Non-analytic processing is used when the task asks for preference (or affective) judgments, whereas analytic processing seems to be the strategy used for recognition tasks. Analytic processing might prevent the feeling of familiarity from occurring as a reference for preference judgments thus enabling positive effects of fluency. In ME studies typically stimuli with a high familiar resemblance are used; consequently, the materials additionally provoke a non-analytic processing strategy [38]. In the current study the opposite might have been the case in the category wood: the wider range of shapes and details might have led to an analytic processing strategy. Further, in our experiments, the participants were instructed after the exposure phase – so until the completion of this phase, the final task (which kind of judgment) was not clear. Thus, probably different processing strategies were used for the two categories of stimuli. Future studies therefore could systematically measure and/or manipulate the stimulus discriminability, test the retrieval of unfamiliar objects under haptic and cross-modal conditions and focus on analytic versus nonanalytic processing strategies. Additionally, other competitive paradigms of preference formation like the “Repeated Evaluation Technique” [40], [41], [42], [43], [44] could be extended to the haptic domain.

Third, the factor complexity influenced the exposure effects in the expected way: liking ratings for high complex stimuli increased in relation to familiarization, whereas ratings for simple stimuli were not affected by the exposure frequencies. This result is in line with Bornstein, Kale and Cornell's [45] findings that simple stimuli showed reduced exposure effects compared to complex stimuli. It was argued that boredom impacts affective judgments in mere exposure experiments. A study using simple versus complex fashion designs (line drawings of dresses), thus more applied materials, reported an complexity×exposure interaction similar to Berlyne's [17] findings by showing liking of complex fashion designs tends to get stronger over exposure time (*n* = 0 to 3 pre-evaluation exposures) [46]. A possible applied implication for the present findings might be in product design: sometimes it is not possible to design a product visually complex (e.g., the shape, the number of elements or edges). A higher variety of haptic cues or haptic information, for example by using various surfaces, might increase the perceived complexity and prone a positive relationship between exposure and liking. Fourth, analysis of the “Need for Touch” data showed effects in participants with a high need for touch, which suggests different sensitivity or saturation levels of MEE. As noted in the introduction, haptic evaluations are affected by the participants' “Need for Touch”: Peck and Childers [37], [47] noted that people with high autotelic “Need for Touch” scores generally prefer tactile feedback in evaluative situations. Especially the positive attitude towards objects increased when haptic elements were present. Further, Peck and Johnson [48], showed that also the persuasion for a product was enhanced through haptic elements within the group of people with high autotelic scores. Our results indicate a similar tendency: seemingly, haptic mere exposure effects occur when people generally believe in the relevance of tactile or haptic cues for their evaluative judgments. This subjective relevance for haptic information as a potential relevant cue for judgments might be the reason for the occurrence of ME effects in the high autotelic group. Possibly, it is harder for low autotelic participants to implement haptic information as a relevant cue for their judgments as they are not used to process information from this modality. Kruglanski, Freund and Bar-Tal [49] suggested that ME effects are more pronounced if a cue is seen as plausible in accordance to the judgment. This relevance hypothesis might also reflect the effects of expertise in the haptic evaluation of objects. Interestingly, Hansen and Bartsch [50] found differences in effect sizes of ME effects for participants with low and high “Personal Need for Structure” (PNS). This personality trait refers to the amount of organization a person prefers to have in general. People with high PNS scores tend to organize their social as well as their non-social environments in a specific way to reduce complexity. Hansen and Bartsch [50] found, that the ME effect was more pronounced when participants scored highly at the PNS scale. It might be possible, that a high “Need for Touch” somehow corresponds with a high “Personal Need for Structure” as “more” (also the haptic) information in a situation might be helpful to organize and structure the environment. Thus, future research could investigate this approach by measuring PNS additionally in order to establish a closer connection between vision and haptics.

Subsuming, it seems that haptic and cross-modal ME effects are influenced by factors similar to those in the visual domain indicating a common cognitive basis. Thus, even after over 40 years of research, the paradigm is still a fascinating and interesting tool to understand processes of evaluation and preference formation and to reveal why we tend to like things we are familiar with.
